# The Adenosinergic System as a Therapeutic Target in the Vasculature: New Ligands and Challenges

**DOI:** 10.3390/molecules22050752

**Published:** 2017-05-06

**Authors:** Joana Beatriz Sousa, Carmen Diniz

**Affiliations:** 1 LAQV/REQUIMTE, Faculty of Pharmacy, University of Porto, 4050-047 Porto, Portugal; joanabeatrizsousa@gmail.com; 2 Laboratory of Pharmacology, Department of Drug Science, Faculty of Pharmacy, University of Porto, 4050-047 Porto, Portugal

**Keywords:** adenosine receptors, nucleoside transporters, vasculature

## Abstract

Adenosine is an adenine base purine with actions as a modulator of neurotransmission, smooth muscle contraction, and immune response in several systems of the human body, including the cardiovascular system. In the vasculature, four P1-receptors or adenosine receptors—A_1_, A_2A_, A_2B_ and A_3_—have been identified. Adenosine receptors are membrane G-protein receptors that trigger their actions through several signaling pathways and present differential affinity requirements. Adenosine is an endogenous ligand whose extracellular levels can reach concentrations high enough to activate the adenosine receptors. This nucleoside is a product of enzymatic breakdown of extra and intracellular adenine nucleotides and also of S-adenosylhomocysteine. Adenosine availability is also dependent on the activity of nucleoside transporters (NTs). The interplay between NTs and adenosine receptors’ activities are debated and a particular attention is given to the paramount importance of the disruption of this interplay in vascular pathophysiology, namely in hypertension., The integration of important functional aspects of individual adenosine receptor pharmacology (such as in vasoconstriction/vasodilation) and morphological features (within the three vascular layers) in vessels will be discussed, hopefully clarifying the importance of adenosine receptors/NTs for modulating peripheral mesenteric vascular resistance. In recent years, an increase interest in purine physiology/pharmacology has led to the development of new ligands for adenosine receptors. Some of them have been patented as having promising therapeutic activities and some have been chosen to undergo on clinical trials. Increased levels of endogenous adenosine near a specific subtype can lead to its activation, constituting an indirect receptor targeting approach either by inhibition of NT or, alternatively, by increasing the activity of enzymes responsible for ATP breakdown. These findings highlight the putative role of adenosinergic players as attractive therapeutic targets for cardiovascular pathologies, namely hypertension, heart failure or stroke. Nevertheless, several aspects are still to be explored, creating new challenges to be addressed in future studies, particularly the development of strategies able to circumvent the predicted side effects of these therapies.

## 1. Introduction

Adenosine is an adenine nucleoside involved in nucleic acid assembly that results from ATP degradation in both the intra- and extracellular environment by the action of specific enzymes, and can act as a signaling molecule by interacting with integral membrane proteins, known as adenosine receptors or purinergic P1-receptors [1]. To date four subtypes have been identified, the adenosine A_1_, A_2A_, A_2B_ and A_3_ receptors. It is established that the intracellular segment of each adenosine receptor subtype interacts with the appropriate heterotrimeric guanine (G) nucleotide-binding protein (G-protein) with subsequent activation of an intracellular signal transduction mechanism. Adenosine receptor subtypes have been grouped into two main categories: (i) subtypes that are coupled to inhibitory G proteins, such as adenosine A_1_ and A_3_ receptors and (ii) subtypes which are coupled to stimulatory G proteins, like the A_2A_ and A_2B_ receptors. Evidence has, however, demonstrated that adenosine receptors are in fact pleiotropic since they may couple with several G proteins/transduction mechanisms depending on their degree of activation or cellular/subcellular localization [2]. Adenosine receptors when activated can lead to interactions with the α, β and γ subunits of the G-protein triggering signaling events [3,4].

In addition to the occurrence of adenosine receptors, adenosine availability is also crucial to discriminate which adenosine receptor subtype is activated. Interstitial levels of adenosine are elevated under conditions of increased metabolic demand (such as exercise) and decreased energy supply (such as ischemia), reaching physiologically relevant concentrations. Adenosine is released into the extracellular space signaling to restore the balance between local energy requirements and energy supply [5]. Released adenosine is quickly transported back into cells by an energy-dependent uptake mechanism, which is part of a purine salvage pathway designed to maintain intracellular ATP levels. Adenosine can be transported from inside to outside the cell and interstitial fluid or vice-versa through specific proteins, the nucleoside transporters (NTs). NTs can, thus, modify extracellular adenosine levels [6,7] since they may facilitate the movement of nucleosides and nucleobases across cell membranes. Transport of adenosine across the cellular membrane is crucial since it contributes to regulate extracellular adenosine levels, and subsequently, adenosine receptor subtype activation. Currently, two types of nucleoside transporters have been identified [8,9]: Equilibrative Nucleoside Transporters (ENT: ENT1, ENT2, ENT3 and ENT4) and Concentrative Transporters (CNT: CNT1, CNT2 and CNT3) [10]. It has been speculated that an increase in the activities of ENT1 and CNT2 may reduce the availability of adenosine to its receptors, conditioning their effects. Thus, NTs act as important players in adenosine function by controlling local levels of adenosine in the vicinity of the adenosine receptors. The effectiveness of this adenosine transport system has been demonstrated to be particularly active in humans, and is responsible for the extremely short half-life of adenosine in human blood.

In addition, adenosine availability also results from ATP enzymatic breakdown of both intra- and extracellular adenine nucleotides and intracellular S-adenosylhomocysteine. The reader is referred to Zimmermann et al. [11], who provide an excellent overview on this complex regulatory system. Briefly, ATP present in the cytosol can be sequentially dephosphorylated to ADP, AMP and then to adenosine. Alternatively, ATP can be released from different types of cells (by exocytosis), and then, metabolized by ectonucleoside triphosphate diphosphohydrolase 1 (ENTPD1 or CD39) to form ADP, AMP and, finally, by AMP hydrolysis to adenosine (via ecto-5′-nucleotidase, NT5E or CD73) [12] and to inosine by adenosine deaminase.

Extracellular disposition of adenosine availability can also be regulated by the presence of guanosine through an unknown mechanism [13], independent of NTs and ectonucleotidase activities. Guanosine increases adenosine and inosine levels [14,15], and can, therefore, alter adenosinergic system dynamics.

All these players—adenosine, adenosine receptors and nucleoside transporters—constitute together the adenosinergic system that, due to the above features, can exert a “fine-tuning” modulation in multiple physiological and pathophysiological processes.

## 2. Adenosine Receptor Ligands and Therapeutic Targets

### 2.1. Adenosine Receptor Structure and Binding “Pocket”

Adenosine receptor structure is crucial for the development of new ligands [16]. Briefly, adenosine receptors present seven transmembrane hydrophobic amino acid domains—TM I-VII—connected by three extracellular and three intracellular hydrophilic loops (with different sizes). These are highly conserved and their residues are crucial for ligand binding/specificity [17,18]. The C-terminal is intracellularly located, on the cytoplasmatic side of the plasma membrane whereas the N-terminal is extracellularly located. The ligand binding site is formed by 3D-arrangement of the transmembrane domains, similar to a “pocket”. The critical interactions required for ligand recognition occur in TM III, V, VI, VII, where two histidine residues are conserved at TM VI, position 52 among adenosine A_1_, A_2A_ and A_2B_ receptors, and were described as crucial for ligand recognition [18,19,20] and contribute to ligand specificity binding within the “pocket”; in adenosine A_3_ receptors, these two histidine residues are lacking (TM VI: His52). Other amino acids residues can also be important for ligand recognition, affinity or binding [19,21,22,23] such as glutamate in TM I of the human adenosine A_2A_ receptor (critically involved in agonist, but not in antagonist, recognition [24]). Adenosine receptors distal region of the second extracellular loop are also involved in agonists and antagonists binding [17]. Indeed, residues negatively charged in adenosine A_2A_ receptor seem to be required for agonists and antagonists binding to the A_2A_ subtype. Additional residues of adenosine A_2A_ receptors were identified in TM V, VI, VII ligand binding [19]: mutation at histidine residues at TM VI, position 52 and TM VII, position 43 in adenosine A_2A_ receptor caused a dramatic loss of ligand affinity; in TM VII, position 43 a substitution of histidine for other residues also decreased ligand affinity [20].

The third intracellular loop of the adenosine A_2A_ receptor seems to be critical for its G-protein selectivity [25]: the cysteine residues forming a disulfide bridge at the third extracellular loop are conserved among the G-protein family and are required for receptor structural integrity and ligand binding [26]. Also the occurrence of mutations in TM IV and of the extracellular loops (both the C-terminal and the N-terminal) of adenosine receptors’ structure have ruled out the importance of this transmembrane domain/ loops in ligand recognition/binding [17,18,27].

A structural feature that should also be considered regarding the development of new adenosine receptor ligands is G-protein coupled receptor dimerization. Adenosine receptors have been described to participate in homo- and/or heterodimerization, and also in oligomerization [28,29] phenomena having a major impact on the pharmacological behavior of those ligands.

### 2.2. New Adenosine Ligands and Their Usefulness

Series of adenosine receptor ligands, both agonists and antagonists [30], have been developed by structure-activity-relationship (SAR) or quantitative-structure-activity-relationship (QSAR) studies, defining both structural and electrostatic requirements for differential ligand affinity of adenosine receptor subtypes. Some of these ligands have been patented and, some are, currently, undergoing clinical evaluation for different therapeutic applications. In Table 1, patents from the last five years related to new adenosine analogues or adenosine receptor antagonists are listed with respective descriptions of the most relevant claimed effects. It is also clear that a recent class of selective adenosine receptor ligands is emerging, the A_2B_ receptor agonists class. Since these selective ligands have become available it has facilitated research on therapeutic applications and knowledge on adenosine receptors modulation [30]. As shown in Table 1, wide therapeutic applications are presented related to several types of pathological conditions and have involved all subtypes of adenosine receptors.

Another important aspect in the development of putative new selective adenosine receptor ligands, particularly of those with specific clinical applications, relies on the widespread actions of adenosine (due to the ubiquitous presence of adenosine and widespread distribution of adenosine receptors in the body), which may contribute greatly to impair safety delivery and clinical effectiveness of a particular compound. Also, the intricacy of adenosine signaling may explain the innumerable side effects reported and, ultimately, the failure of some of the new compounds in phase I, II or III trials.

For example, the compound GW493838, an A_1_ receptor agonist, was tested for its analgesic effect in peripheral nerve injury or neuralgia, and also for its benefic effects on glaucoma and ocular hypertension, but was discontinued. Other similar examples of clinical trials discontinuation have occurred with the adenosine A_1_ antagonists BG9928 and KW-3902 [12], all due to their reported side-effects. Another example is illustrated by the recommendation of the U.S. Food and Drug Administration that the use of regadenoson (CVT-3146, Lexiscan) for cardiac nuclear stress tests of patients with signs or symptoms of unstable angina or cardiovascular instability should be avoided because the drug may increase the risk of a fatal heart attack. Nevertheless, and based on growing scientific evidence, several new adenosine receptor ligands are expected to be approved for clinical use and, hopefully, significantly improve the life style and outcome of patients. In Table 2, examples of ongoing or recently completed clinical trials of adenosine receptor ligands are described. Several clinical applications are reported for the cardiovascular system such as cardiac ischemia, chronic heart failure, atrial fibrillation etc. Nevertheless, in the vasculature few clinical studies have been carried out, “Regadenoson Blood Flow in Type 1 Diabetes” is an example, but further physiological/pharmacological studies in this field are needed to clarify the putative use of adenosine ligands as a therapeutic strategy in the treatment of vascular diseases.

### 2.3. Ligands as Pharmacological Tools

The adenosinergic system has been implicated in several processes such as modulation of neurotransmission, smooth muscle contraction, immune response, both in physiological and pathophysiological conditions. Our knowledge concerning adenosine/adenosine receptor actions/triggering events has improved with the development of ligands, both agonist or antagonists, with individual selectivity for adenosine receptor subtypes. According to the International Union of Pharmacology (IUPHAR), adenosine receptors ligands can be divided into agonists and antagonists depending on their respective adenosine receptor subtype, however there are some studies where some compounds, classified as antagonists, have been described as inverse agonists: caffeine [70] and ZM 241385 [71] as A_2A_ inverse agonists and MRS 1706 [72] as an A_2B_ inverse agonist. A brief summary of the pharmacological ligands currently used for classification of adenosine receptors is presented in Table 3.

Pharmacological studies have revealed that adenosine A_1_ and A_2A_ receptors are high affinity receptors for adenosine although presenting different Kd (A_2A_ receptors require higher concentrations (1–20 nM) than A_1_ receptors (0.3–3 nM) [73]). By contrast, adenosine A_2B_ and A_3_ receptors are low affinity receptors (higher amounts of adenosine are required to activate these subtypes: >1 µM) [74]. An increase in the levels of endogenous adenosine (as a result of NT activity) nearby a specific adenosine receptor subtype can occur leading to its activation. Therefore, the NT may constitute a new target for a different therapeutic approach. Indeed, adenosine mechanisms are the target of commonly used drugs acting by blockade of adenosine reuptake, thus potentiating its actions or antagonizing adenosine receptors. Unfortunately few studies have been carried out and this field of work requires further studies. An approach of an indirect receptor targeting can occur by inhibition of nucleoside transporters. Indeed, nucleoside transporters are a crucial player in adenosine mediated effects by controlling adenosine bioavailability, and subsequently the activation of adenosine receptors [9,75,76]. Evidence also demonstrated that several physiological and pathophysiological conditions [9,75,77,78,79] and hypoxia can also reduce adenosine uptake [80,81] changing adenosine levels nearby adenosine receptors, therefore, conditioning its activation.

Another example of indirect receptor targeting can be achieved by increasing the activity of enzymes responsible for ATP breakdown. Evidence shows that conditions such as inflammation, hypoxia, and stress lead to an increase in ectonucleotidases expression. Moreover, hypoxia can ultimately stimulate CD73 [82,83,84,85], and CD39 [86,87,88,89] and, therefore, increase the ability of the tissue to produce adenosine.

Indirect receptor targeting can be, therefore, an alternative therapeutic strategy using enzymes involved in adenosine production or compounds that modify nucleoside transporter activity as promising therapeutic targets in the cardiovascular disorders. Thus, clinical application of nucleoside transporters can be extended, as at present they have been used successfully in anticancer and antiviral therapy [77,78,129,130].

## 3. Adenosinergic System in the Vasculature

A considerable body of evidence has been gathered in the past years concerning the actions of adenosine in several systems including the cardiovascular system. Indeed, intense research in this field revealed some favorable conditions in which adenosine actions are more relevant. Pathophysiological or hypoxic/ischemic conditions are examples of such, since they favor an augmentation of extracellular adenosine levels with subsequent activation of adenosine receptors.

### 3.1. Vascular Smooth Muscle

Adenosine is able to regulate cardiac functions such as heart rate, contractility and can also influence the coronary flow. Cardiac electrophysiological effects mediated by adenosine occur mostly through direct activation of adenosine A_1_ receptor or, indirectly, by opposing the β-adrenoceptor-mediated effects. A_2A_ receptors have been considered the main receptor subtype involved in coronary blood flow regulation, causing vasodilation in coronary arteries (see reviews [131,132] for further details). Indeed, in vascular tissues, adenosine is known to induce vasodilation, an effect classically ascribed to A_2_ receptors on vascular smooth muscle cells, leading to an increase in blood flow and oxygenation [133]. Nevertheless, evidence has also demonstrated that adenosine A_2_ receptor subtype can mediate vasodilation [134] in an endothelium- and nitric oxide-dependent [131] fashion. Indeed, in more recent studies, it was demonstrated that A_1_ and A_2A_ receptor activation in endothelial cells promotes NO production and, consequently, NO-mediated vasodilation. Adenosine A_2B_ receptors have been described to be involved in the inhibition of vascular smooth muscle cell proliferation and vasodilation in vessels such as aorta and saphenous vein [135,136,137,138,139,140]. On the other hand, A_3_ receptor activation has also been linked to producing relaxation/vasodilation of blood vessels [141].

A broad number of studies reported antimitogenic effects to adenosine, via activation of adenosine A_2B_ receptors in pulmonary [142], aorta [135,143] and glomerular [136] artery smooth muscle cells. Vascular smooth muscle proliferation can be inhibited after adenosine A_2B_ receptor activation through cAMP/Epac (exchange protein directly activated by cAMP) pathway [138]. More recently and, by contrast, adenosine A_1_ receptor was found to promote coronary smooth muscle cells proliferation [139]. These opposite effects ascribed to adenosine in the media layer of arteries, mediated by different adenosine receptor subtypes, evidence the importance of adenosine levels, a crucial factor determining the protective or promoter role of adenosine. Therefore, depending on the subtype of adenosine receptor that is activated, inhibition or stimulation of smooth muscle cells hypertrophy may occur.

### 3.2. Vascular Endothelium

Adenosine may not only promote cell proliferation but can also selectively influence vascular cell death, in a process involving endothelial apoptosis. This process is inhibited by A_2A_ [144] and A_1_ receptor pathways [145]. In addition to endothelial apoptosis, smooth muscle cell apoptosis can also occur due to the action of adenosine via activation of A_2B_ receptor dependent pathways [146].

It is well established that endothelium may influence vascular responsiveness by producing vasoactive substances such as NO, ROS, endothelins and adenosine. For example, in endothelium, it has been described that adenosine induces NO production through adenosine A_1_ and A_2A_ receptor activation pathway which ends with activation of endothelial nitric oxide synthase [147]. Therefore, adenosine can stimulate endothelial NO synthase activity, which in turn, generates higher amounts of NO, a well-known vasodilator [148,149]. Adenosine A_2B_ receptor subtype, in endothelial cells, was implicated in cell proliferation, [150] suggesting that pharmacological or molecular activation of this receptor subtype may be useful in modulating vascular remodeling. Adenosine A_2B_ receptor is, therefore, a protective effector against hyperplasia. Moreover, adenosine A_2B_ receptors were found to be highly expressed in macrophages and vascular smooth muscle cells presenting an important role in the regulation of inflammation and vascular adhesion: deficiency in adenosine A_2B_ receptors was shown to promote lesions or thickness of the neointima after vascular injury [151] revealing its protective role in atherosclerosis. Some studies also identified adenosine A_2A_ [152] and A_3_ [153] receptors as being protective against endothelial injury induced by the inflammatory processes. In vascular tissues, recent studies concerning the role of endothelium in hypertension, had suggested that the main mechanism regulating extracellular adenosine levels involves nucleoside uptake to endothelial cells with the subsequent impairment of adenosine A_1_ receptor activation [154].

### 3.3. Vascular Adventitia

In addition to the adenosine mediated effects, ascribed to receptors/signaling pathways located in smooth muscle cells or endothelium, adenosine actions on sympathetic nerves (or even at central nucleus of the brain) are also of paramount importance in the regulation of vascular tonus. Animal and human studies have demonstrated sustained increases in sympathetic activation and, as a consequence, a direct induction of vascular remodeling. Indeed, sympathetic activation leads to systemic vasoconstriction, increases blood pressure and improves the perfusion pressure. This systemic vasoconstriction could be deleterious to the ischemic organ if not for the simultaneous local inhibitory actions of adenosine, which produces vasodilation and inhibition of noradrenaline release. These actions are, for the most part, circumscribed to the local ischemic tissue so that it is protected from sympathetically mediated vasoconstriction while it benefits from the improved perfusion pressure. Thus, adenosine seems to provide a link between local mechanisms of blood flow autoregulation and systemic mechanisms of autonomic cardiovascular regulation.

Several studies described the occurrence of a neuromodulatory role ascribed to adenosine receptor subtypes activation in sympathetic nerve fibers located in the adventitia layer of pulmonary [155], mesenteric [156,157,158], aorta [159], tail [3,4,154,160,161,162] and renal [163,164] arteries as well as in veins such as mesenteric veins [165,166,167]. For example, adenosine A_2_ receptors, known to facilitate noradrenaline release, may have a profound impact in vascular remodeling, by enhancing noradrenaline levels in the synaptic cleft. On the other hand, the idea that endothelium could influence neurotransmission [155] was recently supported by findings where endogenous adenosine (derived from endothelium) altered neurotransmission (mesenteric and tail arteries) [161]. Endothelium-derived adenosine was also described to activate prejunctional adenosine receptors, mainly A_1_ and A_2A_, which modulate neurotransmission influencing vascular tonus [154]. Taken together, correlated morphological and functional data allowed advances into the insights of neurovascular sympathetic modulation mediated by adenosine receptors, particularly in pathological conditions such as hypertension.

Many studies report greater circulating levels of noradrenaline in patients with hypertension than in normotensive control subjects. In normotensive subjects, increased levels of circulating noradrenaline generally induce a down-regulation of noradrenergic receptors. However, in subjects with hypertension, such down-regulation appears to be missing, resulting in an enhanced sensitivity to noradrenaline. The combination of enhanced sensitivity to and increased circulating levels of noradrenaline likely contributes significantly to sympathetic nervous system activity-related hypertension. In fact, some studies have demonstrated that in hypertensive arteries and veins there are impairment in the neuromodulatory effects mediated by adenosine A_1_ receptors, while the adenosine A_2A_ receptor-mediated facilitation of noradrenaline release is preserved [166,167,168,169]. Adenosine A_2B_ and A_3_ receptors in vessels seem to have an important role in conditions where the amounts of adenosine are higher, i.e., in pathological conditions such as in hypertension [158,170] and diabetes [171,172,173]. Additionally, adenosine A_2B_ receptors increase noradrenaline release [162] while adenosine A_3_ receptors have the opposite effect, inhibiting the release of this neurotransmitter.

An important effect mediated by adenosine in the adventitia layer of vessels is also related with the role of adenosine receptors in inflammation. Indeed, adventitial tissue present several cells involved in inflammatory processes: macrophages, lymphocytes, fibrocytes, cells where adenosine receptor subtypes were found mediating anti or pro-inflammatory effects [174,175,176]. Other type of insights is the interplay between adenosine receptors and signaling molecules involved in inflammation and oxidative stress such as reactive oxygen species (ROS) and NO. Indeed, data indicates that adenosine receptor (A_2A_ subtype) activation promote the increase of ROS generation [177] having a role in oxidative stress and, consequently, in a large number of pathologies where oxidative stress/inflammation is a promotor of the disease. Moreover, adenosine receptors (adenosine A_1_ or A_2A_ receptors) may also activate eNOS leading to an increase of NO production [147], which may impair the deleterious effects mediated by ROS and oxidative stress.

### 3.4. Adenosine Receptors and Angiogenesis

Multiple mechanisms mediated by adenosine lead to the promotion of vessel growth, through stimulation of vascular endothelial cell proliferation, migration and tube formation [178,179]. Adenosine can, thus contribute to angiogenesis and vasculogenesis. The reader is referred to a Carmeliet and Jain article [180] that provides an excellent overview of the angiogenesis process. Numerous studies have shown that adenosine or nucleoside transporter inhibitors can stimulate blood vessel growth [178,181]. Indeed, elevated levels of adenosine can promote the production of pro-angiogenic factors (particularly, vascular endothelial growth factor, VEGF, angiopoietin-1, ANG-1, etc.), key factors to stimulate angiogenesis initiation in several type of cells including endothelial and mesenchymal cells such as monocytes/macrophages. Adenosine has a mitogenic effect on endothelial cells through activation of A_1_, A_2A_ and A_2B_ subtypes [150,179,182]. Hypoxia increases adenosine levels favoring activation of A_2A_ and A_2B_ receptors [183] in parenchymal cells and of A_1_ receptors located in circulating monocytes [184,185], lead to VEGF production. VEGF is, then, able to activate VEGFR2 receptors located in endothelial cells (Tip cell) promoting endothelial cell proliferation, migration and tube formation (key steps of angiogenesis). Additionally, in hypoxic conditions, the expression of adenosine receptor subtypes, A_2A_ and A_2B_ is upregulated, contributing for a favorable environment to the angiogenic process [180]. It is important to notice, however, that adenosine can also modulate the production of anti-angiogenic substances in vascular and immune cells. Adenosine can mediate opposite effects in angiogenesis, by promoting pro-angiogenic or anti-angiogenic factors production. Adenosine can stimulate the release of pro-angiogenic factors such as IL-8, and VEGF, by A_2B_ receptor activation, or can inhibit thrombospondin-1 (anti-angiogenic factor) release by involving A_2A_ receptor subtype dependent pathways [186,187].

### 3.5. Distribution Profile of Adenosine Receptors and NT

The presence of adenosine receptors/nucleoside transporters is, therefore, crucial to predict the impact of adenosinergic system modulation in a particular location. The presence of adenosine receptor subtypes in vascular beds (intima, media and adventitia), both in arteries [3,4,156,161,162,168,169,188,189,190,191,192,193,194,195,196] and veins [197,198,199] has been documented. From immunohistochemical studies it was possible to identify the presence of adenosine A_1_ (tail artery [159], mesenteric artery and vein [166,167,168]), A_2A_ and A_2B_ (tail and mesenteric artery and vein [162,166] and A3 (mesenteric artery and vein [166]) receptors. Recent studies allowed the visualization of adenosine receptor subtypes (A_1_, A_2A_, A_2B_ and A_3_) in sympathetic nerve fibers [167,168,169]. In the endothelium of several arteries such as tail artery [159,162] and aorta [153,159] identification of all adenosine receptors (A_1_, A_2A_, A_2B_ and A_3_) was carried out. Nevertheless, few studies have characterized the presence of adenosine receptors in veins [197,198,199].

Nucleoside transporters presence is also relevant to predict and understand adenosinergic dynamics. Evidence have revealed that CNTs are most likely expressed in a tissue-specific fashion with CNT transport process occurring primarily in specialized epithelia while ENTs present a wide distribution, possibly in all cell types [200]. Nevertheless, studies demonstrated that ENT1 and ENT2, can be found in cell basolateral membranes. ENT2 are also abundantly found in skeletal muscle. ENT3 and ENT4 are widely distributed, but in the heart ENT3 is the most abundant ENT while in the vessels, particularly in endothelium, evidence indicates ENT4 as the most relevant ENT.

In the vasculature, studies on the role of endothelium in hypertension have raised the possibility that the main mechanism regulating extracellular adenosine levels is related with adenosine uptake to endothelial cells, thus, causing a subsequent impairment of adenosine A_1_ receptor activation [161].

The possibility that an increase in the activities of ENT1 and CNT2 may reduce the availability of adenosine to its receptors, conditioning adenosine-mediated effects, have been raised by several authors: For example, King and co-workers [201] have described that ENTs can modulate adenosine-mediated effects in the sinoatrial node of the heart, since dipyridamole potentiates A_1_ receptor-mediated chronotropic effects (via inhibition of adenosine uptake [75]; ENT1/ENT2 modulated adenosine-mediated effects of K^+^ channels and also of the cystic fibrosis transmembrane conductance regulator (CFTR). Other evidence was described in Slc29a1-null mice studies where authors revealed an important role of ENT1 in anxiety-related behavior [202,203] in ethanol preference and consumption [76,204,205] as well as in cardioprotection during ischemia [206]. The later alterations can be ascribed by altered ENT1-mediated modulation of adenosine levels with a subsequently differential adenosine receptor activation and signaling. Consistent with this possibility was the evidence that Slc29a1-null mice have elevated plasma levels of adenosine ^131^.

Nucleoside transporters are relevant players in adenosine functions since they regulate by “fine-tuning” local levels of adenosine in the vicinity of adenosine receptors.

### 3.6. Adenosine Receptors Interaction with P2 Receptors

Evidence has clearly demonstrated that interactions between GPCRs can modulate their activity, by inhibiting or facilitating it. It was also demonstrated that this type of interactions can occur due to receptor dimerization (formation of a physical complex), or due to the occurrence of cross-talk, when second messengers integrate coincident signals from multiple receptors [207,208]. In this regard, purinergic receptors (both P1 and P2) evidence interactions, such as duration, magnitude, and/or direction of the signals triggered by purines or pyrimidines. For instance, adenosine A_1_, A_2A_ receptors or P2X1,3,4,7, or P2Y1,2,4,6,12 subtypes are receptors where such interactions have been reported in several organs (brain [209], kidney [210], oviduct [211], epididymis [212]). In addition, reciprocal influences can also be critical for the effect that each single ligand has on a variety of short- and long term physiological functions [213].

In vascular beds few studies have been done to address the putative interaction between P1 and P2 receptors. For example, regulation of vascular smooth muscle and endothelial cell proliferation by A_2_ receptors and P2Y1 and P2Y2 receptors acting by triggering MAPK pathways has been described [214]; P2X7 and P1 receptors have been linked to apoptosis [215,216]; facilitation of noradrenaline release mediated by A_2A_ receptors is favored by activation of release inhibitory receptors such as P2 but also α_2_-adrenoceptors and A_1_ receptors in tail artery [160]. In arteries and veins, future studies are needed to completely understand the interactions occurring between P1 and P2 receptors, particularly of receptors present in the different vascular layers and of their impact on vascular pathologies.

## 4. Conclusions

In the past years intense research on adenosinergic system dynamics has occurred, enhancing our current knowledge about the interplay between adenosine, adenosine receptors, nucleoside transporters and other signaling molecules and heteroreceptors. The way these interactions are orchestrated in the vasculature, particularly under conditions such as inflammation or oxidative stress, has highlighted the putative role of adenosinergic players as attractive therapeutic targets for several cardiovascular pathologies, namely hypertension, heart failure, stroke, etc.

A renewed interest in this field has led to the development of new adenosine receptor ligands, which is reflected by an increased number of recent patents related to the adenosinergic system. As a consequence, at present several clinical trials are underway, reviewing the potential pharmacotherapy of adenosinergic ligands. In this respect, particularly relevant is the knowledge concerning the presence of adenosine receptors/nucleoside transporters in specific tissue locations since it creates new challenges that can be explored in future studies, namely by elaborating strategies able to circumvent the predicted side effects of these ligands by, for instance, regarding the putative implementation of site/target specific therapies.

## Figures and Tables

**Table 1:** Patents on adenosine receptor ligands: examples from the last 5 years.

Ligands	Claimed Therapeutic Activity	Patent No.	Ref.
Pyridine derivatives	Treatment of stable and unstable angina pectoris and atrial fibrillation	CA2442256C	[31]
Imidazoquinoline derivatives	Therapeutic and/ or preventive treatment of dysfunctions of the heart, kidney, respiratory system, central nervous system.	CA2505910C	[32]
	Treatment of B-cell proliferative disorders	EP2178369A4	[33]
Imidazopyridine derivatives	Treatment, prevention or suppression of diseases and disorders known to be susceptible to improvement by antagonism of the A_2B_ adenosine receptor, such as asthma, chronic obstructive pulmonary disorder, pulmonary fibrosis, emphysema, allergic diseases, inflammation, reperfusion injury, myocardial ischemia, atherosclerosis, hypertension, retinopathy, diabetes mellitus, inflammatory gastrointestinal tract disorders, and/or autoimmune diseases.	US7855202B2	[34]
Pyrazine derivatives	Treatment, prevention or suppression of diseases and disorders known to be susceptible to improvement by antagonism of the A_2B_ adenosine receptor, such as asthma, chronic obstructive pulmonary disorder, pulmonary fibrosis, emphysema, allergic diseases, inflammation, reperfusion injury, myocardial ischemia, atherosclerosis, hypertension, retinopathy, diabetes mellitus, inflammatory gastrointestinal tract disorders, and/or autoimmune diseases.	US785520B2	[35]
Substituted 2-oxy-3,5-dicyano-4aryl-6-aminopyridines	Prophylaxis and/or treatment of various disorders, in particular disorders of the cardiovascular system (cardiovascular disorders), the substances preferably acting as adenosine-receptor selective ligands.	US7855219B2	[36]
Methanocarbacycloakyl nucleoside analogues	Treatment or prevention of various diseases including airway diseases (through A_2B_, A_3_, P2Y2 receptors), cancer (through A_3_, P2 receptors), cardiac arrhythmias (through A_1_ receptors), cardiac ischemia (through A_1_, A_3_ receptors), epilepsy (through A_1_, P2X receptors), and Huntington’s Disease (through A_2A_ receptors).	CA2397366C	[37]
Substituted 2-thio-3,5-dicyano-4-phenyl-6-aminopyridines	Prophylaxis and/or treatment of various diseases such as, for example, diseases of the cardiovascular system, in particular. Suitable active compounds for use in combination are, in particular, active compounds for treating coronary heart diseases, for example nitrates, betablockers, calcium antagonists and diuretics, in particular.	CA2453747C	[38]
Substituted 2-thio-3,5-dicyano-4-phenyl-6-aminopyridines	Treatment of various disorders, i.e., in particular, for example, disorders of the cardiovascular system (cardiovascular disorders). Active compounds suitable for combinations are in particular active compounds for treating coronary heart disease, such as, for example, in particular nitrates, beta blockers, calcium antagonists or diuretics.	CA2469586C	[39]
8-Pyrazolylxanthine derivatives	Treatment of conditions and diseases mediated by the adenosine A_2B_ receptor activity. Such conditions include, but are not limited to, chronic and acute inflammatory diseases involving degranulation of mast cells, e.g., asthma, allergic rhinitis and allergic dermatitis; impaired sensitivity to insulin, e.g., type 2 diabetes, pre-diabetic state, and impaired glucose tolerance; diseases in which angiogenesis is a key component of pathogenesis, e.g., solid tumors and angiogenic retinopathies; apnea of preterm infants; etc.	EP2032797A4	[40]
	Prevention, treatment, or amelioration of cancer, inflammation, auto-immune disease, ischemia-reperfusion injury, epilepsy, sepsis, septic shock, neurodegeneration (including Alzheimer’s Disease), muscle fatigue or muscle cramp (particularly athletes’ cramp).	US20110166093	[41]
(*N*)-Methanocarbaadenine nucleosides	Treatment a number of diseases, for example, inflammation, cardiac ischemia, stroke, asthma, diabetes, and cardiac arrhythmias. The invention also provides compounds that are agonists of both A_1_ and A_3_ adenosine receptors for use in cardioprotection	CN101056879B	[42]
Prodrug derivatives of 2-amino-6-(^13^sulfanyl)-4-(4-{[2,3-dihydroxypropyl]oxy}phenyl)pyridine-3,5-dicarbonitriles	Treatment and/or prophylaxis of diseases, especially of cardiovascular disorders.	EP2379539A1	[43]
	Treating mammals for various disease states, such as gastrointestinal disorders, immunological disorders, hypersensitivity disorders, neurological disorders, and cardiovascular diseases due to both cellular hyperproliferation and apoptosis	US8143249	[44]
Substituted 2-thio-3,5-dicyano-4-aryl-6-aminopyridines	Prophylaxis and/or treatment of various disorders, in particular disorders of the cardiovascular system	CA2440218C	[45]
Xanthine derivatives	Treating mammals for various disease states, such as gastrointestinal disorders, immunological disorders, neurological disorders, and cardiovascular diseases due to both cellular hyperproliferation and apoptosis	CA2524778C	[46]
	Treating asthma, inflammatory gastrointestinal tract disorders, cardiovascular diseases, neurological disorders, and diseases related to undesirable angiogenesis	US20130123280	[47]
	Treating or preventing a cardiovascular disease, a neurological disorder, an ischemic condition, a reperfusion injury, obesity, or wasting disease, or diabetes	US8609833	[48]
Substituted pyrrolopyridine, pyrazolopyridine and isoxazolopyridine derivatives	Treatment and/or prevention of diseases and to their use for preparing medicaments for the treatment and/or prevention of diseases, preferably for the treatment and/or prevention of cardiovascular disorders.	US8609686	[49]
2,4-Disubstituted quinoline derivatives	Treatment of a condition which is treatable by adenosine or an A_3_ agonist	EP2323661B1	[50]
(*N*)-Methanocarbaadenine nucleosides	Treatment a number of diseases, for example, inflammation, cardiac ischemia, stroke, asthma, diabetes, and cardiac arrhythmias	US8518957	[51]
	Preventing, treating, or ameliorating one or more symptoms of glaucoma or ocular hypertension	EP2611502A1	[52]
4-Cycloalkyl- and 4-heterocycloalkyl-3,5-dicyano-2-thio-pyridine derivatives	Treatment and/or prophylaxis of diseases, preferably for the treatment and/or prevention of hypertension and other cardiovascular disorders.	EP2099788B1	[53]
Therapeutic method	Diagnosis and determining effectiveness of treatment of inflammation and in particular to use therefore of biological markers associated with inflammatory states.	US20130345163	[54]
Heteroaryl-substituted dicyanopyridines	Treatment and/or prophylaxis of diseases, preferably for the treatment and/or prevention of cardiovascular disorders.	US8426602	[55]
1*H*-Imidazo-[4,5-*c*]quinolin-4-amine derivatives	Treatment modulation of A_3_ adenosine receptor	US20130197025A1	[56]
Phenylaminothiazole derivatives	Treatment and/or prophylaxis of diseases, preferably for the treatment and/or prevention of hypertension and other cardiovascular disorders.	US8691850	[57]
Substituted 4-amino-3,5-dicyano-2-thiopyridine derivatives	Treatment and/or prophylaxis of diseases, preferably for the treatment and/or prevention of hypertension and other cardiovascular disorders.	US8703934	[58]
Substituted fused pyrimidine	Treating conditions and diseases that are mediated by adenosine receptor activity such as asthma, chronic obstructive pulmonary disorder, angiogenesis, pulmonary fibrosis, emphysema, allergic diseases, inflammation, reperfusion injury, myocardial ischemia, atherosclerosis, hypertension, congestive heart failure, retinopathy, diabetes mellitus, obesity, inflammatory gastrointestinal tract disorders, and/or autoimmune diseases	US8796290B2	[59]
Fused pyrimidine compounds	Treating conditions and diseases that are mediated by adenosine receptor activity. These compounds are useful in the treatment, prevention or suppression of diseases and disorders that may be susceptible to improvement by antagonism of the adenosine receptor, such as asthma, chronic obstructive pulmonary disorder, angiogenesis, pulmonary fibrosis, emphysema, allergic diseases, inflammation, reperfusion injury, myocardial ischemia, atherosclerosis, hypertension, congestive heart failure, retinopathy, diabetes mellitus, obesity, inflammatory gastrointestinal tract disorders, and/or autoimmune diseases	CA2718983C	[60]
Substituted 2,4′- and 3,4′-bipyridine derivatives	Treatment and/or prophylaxis of diseases, preferably for the treatment and/or prevention of hypertension and other cardiovascular disorders	CA2662728C	[61]
2-Alkoxy-substituted dicyanopyridines	Treatment and/or prophylaxis of diseases, preferably for the treatment and/or prevention of cardiovascular disorders.	US9205077	[62]
2-amino-6-({[2-(4-chlorophenyl)-1,3-oxazol-4-yl]methyl}sulfanyl)-4-(4-{[2,3-dihydroxypropyl]oxy}phenyl)pyridine-3,5-dicarbonitrile	Treatment and/or prophylaxis of diseases, and their use for the manufacture of medicaments for the treatment and/or prophylaxis of diseases, especially of cardiovascular disorders.	US8741834	[63]
2-Amino-6-({[2-(4-chlorophenyl)-1,3-thiazol-4-yl]methyl}thio)-4-[4-(2-hydroxyethoxy)phenyl]pyridine-3,5-dicarbonitrile	Treatment and/or prophylaxis of diseases, and their use for the manufacture of medicaments for the treatment and/or prophylaxis of diseases, especially of cardiovascular disorders.	CA2695036C	[64]
Substituted aryloxazole derivatives	Treatment and/or prophylaxis of diseases, and their use for the manufacture of medicaments for the treatment and/or prophylaxis of diseases, especially of cardiovascular disorders.	US9095582	[65]
Substituted 8-[6-carbonylamino-3-pyridyl] xanthines	Therapeutic methods are provided herein for treating a pathological condition or symptom in a mammal, such as a human, wherein the activity, e.g., over- activity, of adenosine a_2b_ receptors is implicated in one or more symptoms of the pathology and antagonism (i.e., blocking) is desired to ameliorate such symptoms.	WO2011005871A1	[66]
2-Chloro-*N*^6^-(3-iodobenzyl)-adenosine-5′-*N*-methyluronamide (Cl-IB-MECA)	Treatment of hepatocellular carcinoma	US20150018299	[67]
Substituted fused pyrimidine compounds	Treatment, prevention or suppression of diseases and disorders that may be susceptible to improvement by antagonism of the adenosine receptor, such as asthma, chronic obstructive pulmonary disorder, angiogenesis, pulmonary fibrosis, emphysema, allergic diseases, inflammation, reperfusion injury, myocardial ischemia, atherosclerosis, hypertension, congestive heart failure, retinopathy, diabetes mellitus, obesity, inflammatory gastrointestinal tract disorders, and/or autoimmune diseases	US9284316	[68]
	Treating conditions and diseases that are mediated by thereof as A_2B_ adenosine receptor antagonists	CN103261200B	[69]

**Table 2:** Clinical trials of adenosine receptor ligands: example of recently completed or ongoing studies.

Target	Ligands	Clinical Trials: Study	C. T. Identifier Code	Ref.
All adenosine receptor subtypes	Agonist: adenosine	Prophylactic Intra-coronary Adenosine to Prevent Post Coronary Artery Stenting Myonecrosis	NCT00612521	[90]
		Circulating Adenosine Levels Before and After Intravenous (IV) Persantine	NCT00760708	[91]
All adenosine receptor subtypes	Antagonist: caffeine	Caffeine for Motor Manifestations of Parkinson’s Disease	NCT01190735	[92]
		Caffeine for Excessive Daytime Somnolence in Parkinson’s Disease	NCT00459420	[93]
		The Impact of Caffeine on Brachial Endothelial Function in Healthy Subjects and in Patients With Ischemic Heart Disease	NCT00564824	[94]
		Caffeine as a Therapy for Parkinson’s Disease	NCT01738178	[95]
Adenosine A1 receptor	Agonist: BAY1067197	Multiple Dose Study in Heart Failure of BAY 1067197 (PARSiFAL)	NCT02040233	[96]
		Study to Assess the Safety of BAY1067197 in Stable Heart Failure Patients on Standard Therapy Including ß-blocker	NCT01945606	[97]
		A Trial to Study Neladenoson Bialanate Over 20 Weeks in Patients With Chronic Heart Failure With Reduced Ejection Fraction (PANTHEON)	NCT02992288	[98]
Adenosine A1 receptor	Agonist: tecadenoson	Safety Study of Tecadenoson to Treat Atrial Fibrillation	NCT00713401	[99]
Adenosine A1 receptor	Antagonist: PBF-680	“First-in-human” Study To Assess the Safety and Tolerability of PBF-680 in Male Healthy Volunteers	NCT01845181	[100]
		A Study to Assess the Efficacy of a 5-day, 10-mg PBF-680 Oral Administration on Late Asthmatic Responses (LAR) in Mild to Moderate Asthmatic Patients.	NCT02635945	[101]
		Study to Assess the Efficacy of a Single PBF-680 Oral Administration to Attenuate Adenosine 5′-Monophosphate Challenge-induced Airway Hyperresponsiveness in Mild-to-moderate Asthmatics	NCT01939587	[102]
Adenosine A2A receptor	Agonist: regadenoson	Adenosine 2A Agonist Lexiscan in Children and Adults With Sickle Cell Disease	NCT01085201	[103]
		ADVANCE MPI 2: Study of Regadenoson Versus Adenoscan^®^ in Patients Undergoing Myocardial Perfusion Imaging (MPI)	NCT00208312	[104]
		Myocardial Perfusion Magnetic Resonance Imaging Using Regadenoson	NCT00881218	[105]
		Regadenoson Blood Flow in Type 1 Diabetes (RABIT1D) (RABIT1D)	NCT01019486	[106]
		A Phase II Trial of Regadenoson in Sickle Cell Anemia	NCT01788631	[107]
Adenosine A2A receptor	Agonist: binodenoson	Efficacy and Safety Study of Binodenoson in Assessing Cardiac Ischemia (VISION-305)	NCT00944970	[108]
Adenosine A2A receptor	Agonist: MRE0094	Safety and Efficacy Study of MRE0094 to Treat Chronic, Neuropathic, Diabetic Foot Ulcers	NCT00312364	[109]
Adenosine A2A receptor	Antagonist: preladenant	A Study to Assess Pharmacokinetics of Preladenant in Participants With Chronic Hepatic Impairment (P06513)	NCT01465412	[110]
		Placebo Controlled Study of Preladenant in Participants With Moderate to Severe Parkinson’s Disease (P07037)	NCT01227265	[111]
		A Dose Finding Study of Preladenant (SCH 420814) for the Treatment of Parkinson’s Disease (PD) in Japanese Patients (P06402)	NCT01294800	[112]
		Study of Preladenant for the Treatment of Antipsychotic Induced Movement Disorders in Participants With Schizophrenia (Study P04628)	NCT00686699	[113]
		Study of Preladenant for the Treatment of Neuroleptic Induced Akathisia (Study P05145AM1) (COMPLETE)	NCT00693472	[114]
		A Placebo- and Active-Controlled Study of Preladenant in Early Parkinson′s Disease (PD) (P05664) (PARADYSE)	NCT01155479	[115]
Adenosine A2A receptor	Antagonist: istradefylline	Effect of Mild Hepatic Impairment on the Pharmacokinetics of Istradefylline	NCT02256033	[116]
		A 12-week Randomized Study to Evaluate Oral Istradefylline in Subjects With Moderate to Severe Parkinson′s Disease (KW-6002)	NCT01968031	[117]
		Long Term Study of Istradefylline in Subjects With Moderate to Severe Parkinson′s Disease	NCT02610231	[118]
		Study of Istradefylline (KW-6002) for the Treatment of Restless Legs Syndrome	NCT00199446	[119]
Adenosine A3 receptor	Agonist: CF101	Trial of CF101 to Treat Patients With Psoriasis	NCT00428974	[120]
		Oral CF101 Tablets and Methotrexate Treatment in Rheumatoid Arthritis Patients	NCT00556894	[121]
		Safety and Efficacy of Daily CF101 Administered Orally in Subjects With Elevated Intraocular Pressure	NCT01033422	[122]
		Trial of CF101 to Treat Patients With Dry Eye Disease	NCT01235234	[123]
		Safety and Efficacy Study of CF101 to Treat Keratoconjunctivitis Sicca	NCT00349466	[124]
Adenosine A3 receptor	Agonist: CF102	A Phase 1–2 Study of CF102 in Patients With Advanced Hepatocellular Carcinoma	NCT00790218	[125]
		A Phase 1/2 Study of CF102 in Patients With Chronic Hepatitis C Genotype 1	NCT00790673	[126]
		Phase 2, Randomized, Double-Blind, Placebo-Controlled of the Efficacy and Safety of CF102 in Hepatocellular Carcinoma (HCC)	NCT02128958	[127]
Adenosine A3 receptor	Antagonist: PBF-677	“First-in-human” Study To Assess the Safety and Tolerability of PBF-677 in Healthy Volunteers	NCT02639975	[128]

**Table 3:** Ligands currently used for adenosine receptors classification.

Ligand Type	Abbrev.	Ligand	Adenosine Receptor Subtype
AGONIST	ADO	Adenosine	A_1_, A_2A_, A_2B_, A_3_
	NECA	5′-*N*-Ethylcarboxamidoadenosine	A_1_, A_2A_, A_2B_, A_3_
	CPA	*N*^6^-Cyclopentyladenosine	A_1_
	CCPA	2-Chloro-CPA	A_1_
	CGS 21680	2-*p*-(2-Carboxyethyl)phenethylamino-5′-*N*-ethylcarboxamidoadenosine hydrochloride	A_2A_
	IB-MECA	1-Deoxy-1-[6-[[(3-iodophenyl)methyl]amino]-9*H*-purin-9-yl]-*N*-methyl-*b*-d-ribofuranuronamide	A_3_
	2Cl-IB-MECA	2-Chloro-*N*^6^-(3-iodobenzyl)-5′-(*N*-methylcarbamoyl)adenosine	A_3_
ANTAGONIST	Teophylline	3,7-Dihydro-1,3-dimethyl-1*H*-purine-2,6-dione	A_1_, A_2A_, A_2B_, A_3_
	Caffeine	1,3,7-Trimethylpurine-2,6-dione	A_1_, A_2A_
	DPCPX	1,3-Dipropyl-8-cyclopentylxanthine	A_1_
	SCH 58261	5-Amino-7-(2-phenylethyl)-2-(2-furyl)-pyrazolo-[4,3-*e*]-1,2,4-triazolo[1,5-*c*]pyrimidine	A_2A_
	ZM 241385	4-(2-[7-Amino-2-[2-furyl]-[1,2,4]triazolo[2,3-*a*][1,3,5]triazin-5-yl-amino]ethyl)phenol	A_2A_
	MRS 1754	*N*-(4-Cyanophenyl)-2-[4-(2,6-dioxo-1,3-dipropyl-2,3,4,5,6,7-hexahydro-1*H*-purin-8-yl)-phenoxy]acetamide	A_2B_
	MRS 1706	*N*-(4-Acetylphenyl)-2-[4-(2,3,6,7-tetrahydro-2,6-dioxo-1,3-dipropyl-1H-purin-8-yl)phenoxy]acetamide	A_2B_
	MRS 1220	*N*-[9-Chloro-2-(2-furanyl)[1,2,4]-triazolo[1,5-*c*]quinazolin-5-yl]benzene acetamide	A_3_
	MRS 1523	2,3-Diethyl-4,5-dipropyl-6-phenylpyridine-3-thiocarboxylate-5-carboxylate	A_3_
